# Molecular footprint of *Frankliniella occidentalis* from India: a vector of Tospoviruses

**DOI:** 10.1080/23802359.2018.1536446

**Published:** 2018-11-25

**Authors:** Devkant Singha, Vishal Kumar V, Rajasree Chakraborty, Shantanu Kundu, Arunkumar Hosamani, Vikas Kumar, Kaomud Tyagi

**Affiliations:** a Centre for DNA Taxonomy, Molecular Systematic Division, Zoological Survey of India, New Alipore, Kolkata, India;; b University of Agricultural Sciences, Raichur, Karnataka, India

**Keywords:** Thrips, invasive pest, DNA barcoding, quarantine regulation, ecosystem

## Abstract

The western flower thrips, *F. occidentalis* is a vector of Tospoviruses and native to Western North America and Mexico. The present study is based on collected *F. occidentalis* specimens from Karnataka state in southern India and morphologically identified through available keys. The generated DNA barcode data show 99–100% similarity with the database sequences of *F. occidentalis*. The phylogenetic analysis (NJ, ML, and BA) shows three distinct clades of *F. occidentalis* in the present dataset with high bootstrap supports and posterior probabilities. The K2P genetic distances further depicted high similarity of the generated sequences from India and Netherlands. The Clade-1 (India + Netherlands) also shows a close relationship with Clade-2 (Kenya) rather than Clade-3 (Canada + USA). This study recorded the first genetic footprint of *F. occidentalis* in India and indicated the gene flow from the Netherlands to India. The similar molecular techniques may help to detect the invasion of many alien species in the near future and assists the quarantine regulations to protect the native ecosystem.

## 1. Introduction

The small, minute organism with fringed wing insects are known as thrips and belong to order Thysanoptera. Globally, near about 6154 species are reported, out of these, only 1% are reported as pests and vectors of Tospoviruses (ThripsWiki 2018). Among global thrips diversity, India shares 12% with 739 species under 5 families (Tyagi and Kumar 2016). Thrips are the sole transmitters of Tospoviruses and causing plant disease in several plant families across different zoogeography. Till now, only 15 species are known as vectors of plant Tospoviruses (family Bunyaviridae) (Riley et al. 2011, Zhou and Tzanetakis 2013). Thus, concerning their impact on the global agricultural economy, accurate species identification is the basic need for implementation of effective integrated pest management strategies.

The western flower thrips, *F. occidentalis* was native to Western North America and Mexico (Mound and Marullo 1996). In the recent past, the species has turned out to be one of the major pests worldwide by making a severe economic loss in many agricultural and horticultural crops (Wijkamp et al. 1995; Kirk 2002; Kirk and Terry 2003; Riley et al. 2011). This species can transmit five different Tospovirus strains, viz., *Tomato spotted wilt virus, Impatiens necrotic spot virus*, *Groundnut ringspot virus*, *Chrysanthemum stem necrosis virus*, and *Tomato chlorotic spot virus* (Whitfield et al. 2005). Globally, around $1 billion economic loss was estimated by a single Tospovirus, TSWV and $50 million loss from Netherland (Rugman-Jones et al. 2010). Further, the rigorous estimation revealed that a total of $326 million losses was detected by Tospoviruses from 1996 to 2006 in Georgia (Riley et al. 2011). Recently, *F. occidentalis* was morphologically detected from Karnataka state in southern India (Tyagi and Kumar 2015). This species exhibits color polymorphism, ranging from yellow, bicolored to brown which are morphologically similar but genetically different (Rugman-Jones et al. 2010). The cryptic behavior, sexual dimorphism, and polymorphism phenomenon are common in thrips species (Tyagi et al. 2017) that required molecular interventions for accurate species identification and delimitations. Further, DNA barcoding studies revealed multiple cryptic species in *Frankliniella schultzei* (Tyagi et al. 2017). Thus, the present study was aimed to determine the identity of *F. occidentalis* through molecular data to adjudicate the possible invasion.

## 2. Materials and methods

### 2.1. Sampling, vouchering and molecular assessment

**Figure 1.**
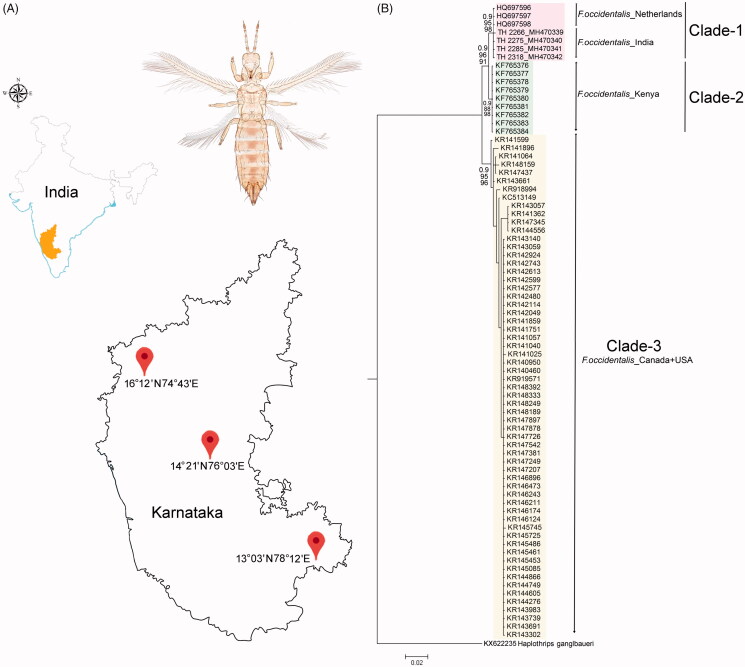
(A) Map showing the collection localities of *F. occidentalis* in Karnataka states of southern India with latitude and longitude. (B) Bayesian (BA) phylogeny of the studied *F. occidentalis* species. The BA posterior probability, and bootstrap support resulted in NJ and ML tree were superimposed with each nodes. The collection localities of the studied and database sequences were incorporated with the voucher IDs and accession numbers in the phylogeny. Colour bars show the genetic variability and distinct clades of the *F. occidentalis* population.

Total 11 specimens of *F. occidentalis* were collected from three different localities (16°12′N 74°43′E, 14°21′N 76°03′E, 13°03′N 78°12′E) in Karnataka state of southern India (Figure 1(A)). The specimens were collected by standard beating methods from different host plants (bitter gourd, watermelon, cucumber, and pumpkin) and preserved in 70% ethanol and subsequently stored at −20 °C for DNA analysis. After non-destructive DNA isolation, the specimens were retrieved and mounted onto glass slides in Canada balsam for morphological identification. The specimens were identified by available morphological keys (Mound and Marullo 1996; Cavalleri and Mound 2012). The photographs were taken through a Leica Trinocular Microscope (Leica DM-1000) and using a Leica software application suite (LAS EZ 2.1.0). The voucher specimens were deposited in National Zoological Collections of Zoological Survey of India with the sample code and registration No. TH-2266 (9310/H17), TH-2275 (9314/H17), TH-2285 (9313/H17), TH-2318 (9315/H17), TH-2230 (9311/H17), TH-2314 (9312/H17), TH-2340 (9391/H17), TH-2339 (9392/H17), TH-2338 (9393/H17), TH-2336 (9422/H17), and TH-2337 (9433/H17).

The genomic DNA was extracted using the QIAamp DNA Mini Kit (Qiagen, Valencia, CA). An intersegmental abdominal cut was made to only four specimens and lysed overnight at 56 °C in buffer ATL with proteinase-K. The polymerase chain reaction (PCR) was performed to amplify the mitochondrial DNA barcode gene segment (∼648 bp) for molecular-based species identification. The published primer pair, HCO-2198: 5′-TAAACTTCAGGGTGACCAAAAAATCA-3′ and LCO-1490: 5′-GGTCAACAAATCATAAAGATATTGG-3′ was used to amplify the targeted mitochondrial region (Folmer et al. 1994). The total volume of PCR reaction was 30 μl containing 20 Picomoles of each primer, 20 mM Tris-HCl (pH 8.0), 100 mM KCl, 0.1 mM EDTA, 1 mM DTT, 1.8 mM MgCl2, 0.25 mM of each dNTP, and 1U of Taq polymerase (Takara BIO Inc., Shiga, Japan) with the following thermal profile: 5 min at 94 °C; followed by 40 cycles of 30 s at 94 °C, 40 s at 49 °C, 1 min at 72 °C, and final extension for 5 min at 72 °C. The PCR products were checked in 1% agarose gel, stained with ethidium bromide (10μg/mL). The PCR products were purified using the QIAquick Gel Extraction Kit (Qiagen) following the manufacturer’s protocols. The cycle sequencing of the purified PCR products was performed with BigDye^®^Terminator ver. 3.1 Cycle Sequencing Kit (Applied Biosystems, Inc. California, US) by 48 capillary ABI 3730 Genetic analyzer, housed at the Zoological Survey of India, Kolkata.

### 2.2. Sequence analysis and dataset preparation

The bi-directional chromatograms of each specimen were checked in MEGA6 (Tamura et al. 2013) and the noisy parts were trimmed at both the ends. Each sequence was evaluated in the nucleotide BLAST (BLASTn) online program (https://blast.ncbi.nlm.nih.gov/Blast.cgi) and ORF finder (http://www.ncbi.nlm.nih.gov/gorf/gorf.html) to examine the complete alignment and stop codons. Primarily, the generated sequences were identified in the online identification system, in GenBank with ‘Highly similar sequences (megablast)’ and BOLD databases with ‘All Barcode Records on BOLD’. Based on the similarity search and considering the sequence length (>500bp), total 73 sequences of *F. occidentalis* were acquired from the GenBank database to make a final dataset. The final dataset of 77 sequences was aligned using ClustalX software and finally, 514 bp of mtCOI were adopted for estimating genetic divergence and phylogeny (Thompson et al. 1997). The sequence of *Haplothrips ganglbaueri* (KX622235) was further incorporated in the dataset as an out-group.

### 2.3. Genetic divergence, haplotypes, and phylogeny

The genetic divergence was calculated by Kimura 2 parameter (K2P) in MEGA6 (Tamura et al. 2013). The model selection was based on the Bayesian Information Criterion (BIC) computed by PartitionFinder version 1.1.1 (Lanfear et al. 2012). The best fit model GTR + I + G (NST = 6) was selected for both ML and BA phylogeny. The Neighbor-Joining (NJ), Maximum Likelihood (ML), and Bayesian analysis (BA) were implemented to test the reciprocal monophyletic criteria for species identification. The NJ and ML tree were performed under the optimality criteria by using PAUP 4.0b10 (Swofford 2002) with 1000 bootstrap support. For BA, Markov Chain Monte Carlo (MCMC) was performed with four chains for 1,000,000 generations, with trees sampled every 100 generations (the first 1000 trees were discarded as ‘burn-in’) using MrBayes 3.1 (Ronquist and Huelsenbeck 2003). MCMC analysis was stationary when the maximum standard deviation of split frequencies reached below 0.01 and potential scale reduction factor approached 1.0.

## 3. Results and discussion

The collected thrips specimens were identified as *F. occidentalis* (female) with the following diagnostic characters: body pale yellow with brown patches on abdominal tergites; head with three pairs of ocellar setae, pair III situated between fore and hind ocelli; postocular setae IV longer than others. Eyes without pigmented facets. Antenna with 8-segments with simple pedicle on segment III, segments III and IV each with forked sense cone. Pronotum with two to four small setae between the major anteromarginal setae. Mesonotal surface with faint transverse line, anteromedian campaniform sensilla present. Metanotal median setae situated at the anterior margin, with irregular reticulations postero-medially and paired campaniform sensilla. Fore wing first vein and second vein with complete rows of setae. Abdominal tergite VIII with an irregular comb of microtrichia at their posterior margin. Tergite IX S1 setae longer than tergite X.

DNA barcoding is evidenced as an effective molecular tool for (i) species identification (Hebert et al. 2003; Iftikhar et al. 2016; Laskar et al. 2018), (ii) recognition of the species complexes and cryptic diversity (Tyagi et al. 2017; Kundu et al. 2018), (iii) estimation of the phylogeny and evolutionary relationship of species (Buckman et al. 2013; Kundu et al. 2016; Tyagi et al. 2017; Laskar et al. 2018), and (iv) detection of invasive species and their probable route (Tyagi et al. 2015; Kundu et al. 2016) and resolve several biological questions in a wide variety of animal taxa. The generated DNA barcode sequences of the four specimens were annotated (616bp) and submitted into the global database (GenBank) to acquire the unique accession numbers (TH-2266: MH470339, TH-2275: MH470340, TH-2285: MH470341, and TH-2318: MH470342). The sequence similarity search in both BLASTn and BOLD-IDs revealed definitive identity matches (99–100%) for all four generated sequences with the database sequences of *F. occidentalis*. The estimated phylogenetic tree (NJ, ML, and BA) depicted cohesive clustering of the identified *F. occidentalis* sequences with the database sequences with high bootstrap support and posterior probabilities (Figure 1). The phylogenetic analysis shows three distinct clades of *F. occidentalis* in the present dataset: Clade-1, Clade 2, and Clade-3 (Figure 1(B)). The Clade-1 shows the cohesive clustering of the *F. occidentalis* sequences, generated from India with the database sequences from the Netherlands. Further, the Clade-2 and Clade-3 represented by the database sequences of *F. occidentalis* generated from Kenya and Canada respectively. One database sequence, generated from the USA, shows cladding with the Clade-3. The present phylogeny also depicted the close relationship of Clade-1 (India + Netherlands) with Clade-2 (Kenya) rather than Clade-3 (Canada + USA) (Figure 1(B)). The overall mean genetic distance within the studied dataset of *F. occidentalis* was 1.5% ranging from 0% to 4.7%. The *F. occidentalis* sequences generated from Canada and India shows 0.4% and 0.1% conspecific genetic divergence respectively. Further, the *F. occidentalis* sequences generated from the Netherlands and Kenya shows 0% genetic divergence within the species and cannot be calculated for a single sequence generated from the USA. The generated sequences of *F. occidentalis* from India is identical in the genetic distance (0%) with the sequences generated from the Netherlands. Further, the Clade-1 (India + Netherlands) shows 1–3.9% genetic divergence with other *F. occidentalis* sequences generated from Kenya (Clade-2), and Canada + USA (Clade-3). (Table 1).

**Table 1.** The K2P genetic divergence over sequence pairs between and within groups of the studied *F. occidentalis* species.

Population	Genetic divergence (%)				
	Between populations				Within populations
*F. occidentalis*_Canada					0.4
*F. occidentalis*_USA	0.5				n/c
*F. occidentalis*_Kenya	3.6	3.2			0
*F. occidentalis*_Netherlands	3.8	3.4	1.0		0
*F. occidentalis*_India	3.9	3.5	1.0	0	0.1

n/c: not able to calculate due to single sequence.

Hence, based on the molecular data, the present study assumed the flow of genetic material, and the European country (Netherlands) may be a probable source of invasion of *F. occidentalis* to India (South Asian countries). The genetic distance and phylogeny revealed the close relationship of European and South Asian populations of *F. occidentalis* with the African populations. The invasion of biological taxa in the non-native ecosystem is well-known biotic threats for indigenous animal or plant species. Furthermore, the trading of agricultural resources within and between the different countries facilitates to maintain economic growth. However, a thorough inspection is necessitated while trading of any agricultural resources and possible associated organisms to mitigate the invasion of alien species and protect the ecosystem. In this circumstance, the concern quarantine agencies may adopt the molecular tools for detecting the organisms at any life stages or at any forms. In the recent past, scientists have evidenced the invasion of the serious pest species of Papaya, *Thrips parvispinus* from Indonesia to India (Tyagi et al. 2015). In this study, we recorded the genetic footprint of another serious pest and vector species, *F. occidentalis* from India. In conclusion, more molecular data of this serious pest species from different geographical regions and their population genetics may be helpful to trace the exact route of invasion.
